# Longistyline C acts antidepressant in vivo and neuroprotection in vitro against glutamate-induced cytotoxicity by regulating NMDAR/NR2B-ERK pathway in PC12 cells

**DOI:** 10.1371/journal.pone.0183702

**Published:** 2017-09-05

**Authors:** Yamin Liu, Ning Zhao, Chenchen Li, Qi Chang, Xinmin Liu, Yonghong Liao, Ruile Pan

**Affiliations:** Institute of Medicinal Plant Development, Chinese Academy of Medical Science, Peking Union Medical College, Beijing, China; Hokkaido Daigaku, JAPAN

## Abstract

Depressive disorder is a common psychiatric disease which ranks among the leading cause of disability worldwide. The antidepressants presently used had low cure rate and caused a variety of side-effects. The screening of antidepressant drugs is usually used classic behavioural tests and neuroprotective strategy. Longistyline C, a natural stilbene isolated from the leaves of Cajanuscajan (L.) Millsp, was firstly investigated the antidepressant effect using animal behavioural tests, and studied the neuroprotection and its possible signaling pathways on glutamate-induced injury in PC12 cells. The results of animal test demonstrated that longistyline C had the antidepressant activity, which the effect is similar to the positive control. In current study, we investigated the effect of longistyline C on glutamate-induced injury in PC12 cells and explored its possible signaling pathways. The results demonstrated that pretreatment with longistyline C at the concentrations of 2–8 μmol/L for 24 h had a significant reduction of the cytotoxicity induced by glutamate (15 mmol/L) in PC12 cells using MTT, lactate dehydrogenase (LDH) release assay and Annexin V—PI double staining. Subsequently, we found that pretreatment with longistyline C (8 μmol/L) could drastically down-regulate the over-expression of NMDAR/NR2B and Ca^2+^/calmodulin-dependent protein kinase II (CaMKII), up-regulate the expressions of p-ERK and p-CREB and alleviate ER stress. In conclusison, longistyline C is most possibly through regulating NMDAR/NR2B-ERK1/2 related pathway and restoring endoplasmic reticulum function to exert neuroprotective effect against glutamate-induced injury in PC12 cells.

## Introduction

Depressive disorder (MD) is a common psychiatric disease which ranks among the leading cause of disability worldwide [1]. The antidepressants presently used are mainly based on monoamine neuro-transmitters hypothesis, which the cure rate is as low as 70% approximately, and associated with many serious adverse effects, such as cardiac toxicity, sexual dysfunction, body weight gain and sleep disorder [2, 3]. Thus, a safer, better-tolerated drug with higher effect is still on the way.

With the development of neuroscience, antidepressant research has transferred beyond the monoamine hypothesis to other pathological processes, such as stress and neuroplasticity [4, 5]. Exposure to psychological and physiological-related chronic stress is one cause of the activation of the hypothalamic—pituitary—adrenal (HPA) axis, which initiates a sustained rise in serum glucocorticoids. It has been proved that high serum glucocorticoids will increase the synthesis of excitatory glutamates, and decrease the level of inhibitory gamma-aminobutyric acid (GABA), thus change the relative ratio of excitatory and inhibitory signal [6–8]. Moreover, the glutamate concentration in the synaptic gaps will rise by the way of reducing the reuptake of glutamate on the stress situation [9]. Excessive glutamate leads to sustained activation of N-methyl-D-aspartic (NMDA)-type glutamate receptors, triggering a sequence of events initiated by cytosolic influx of calcium ions (Ca^2+^), subsequently causing reactive oxygen species (ROS) increase, resulting in neuronal damage and cell death [10–12]. Therefore, screening of antidepressant drugs is not only used classic behavioural tests such as the tail suspension and forced swim [13, 14], but also applied neuroprotective strategy such as nerve cell damage models induced by glutamate or corticosterone [15, 16].

**Fig 1:**
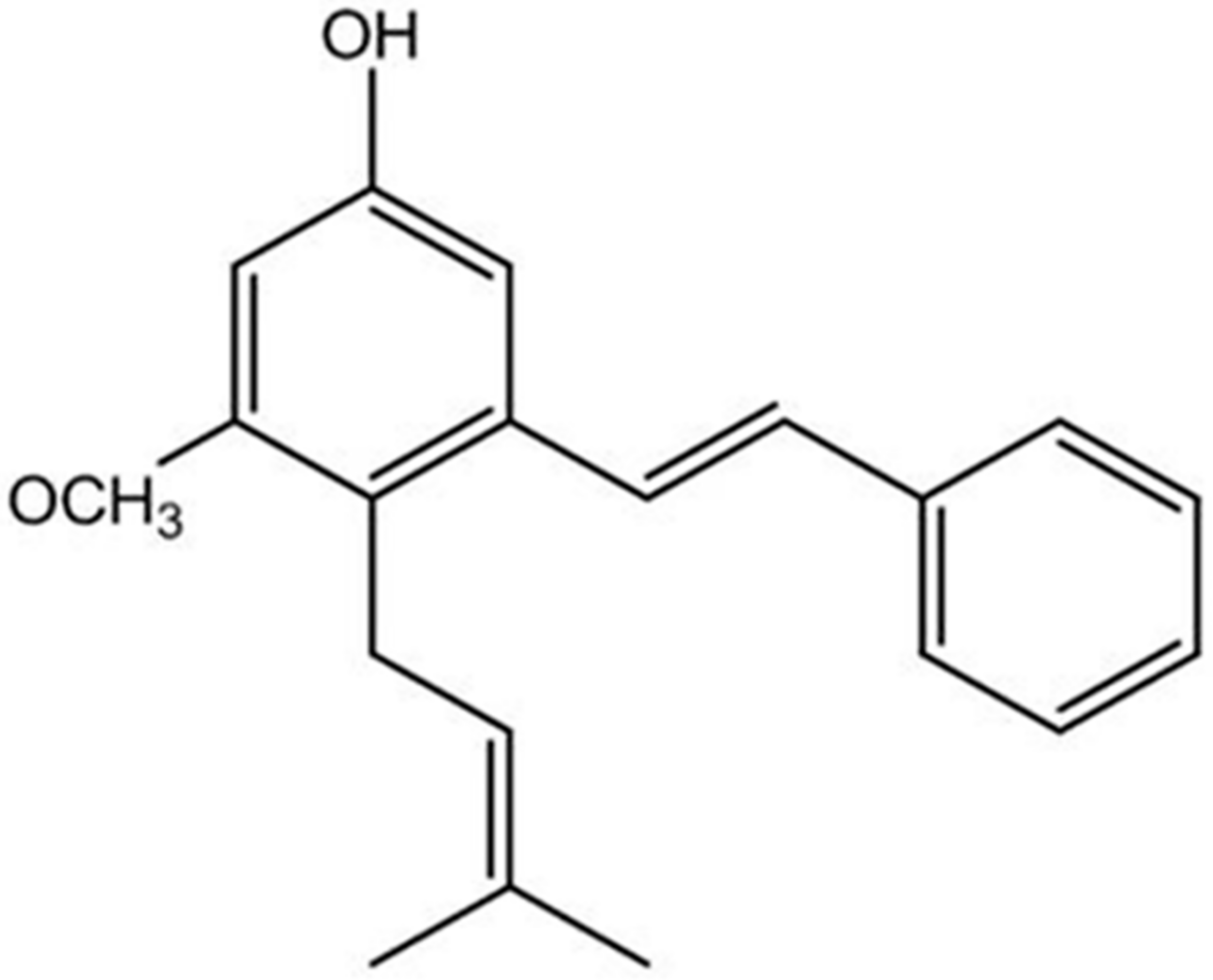
The structure of longistyline C.

Stilbenes are a class of plant polyphenols and have attracted intense interest for their diverse biological activities. Longistyline C (3-hydroxy-6-prenyl-5- methoxystilbene, Fig 1), a natural stilbene isolated from the leaves of *Cajanuscajan* (pigeonpea, family Leguminosae) is proved to have neuroprotection against glutamate-induced injury in PC12 cells in a dose-dependent manner [17]. However, little was known about its antidepressant effect using animal behavioural tests and the underlying neuroprotective mechanisms of longistyline C.

The present study aimed to examine the in vivo antidepressant effect of longistyline C using behavioural tests, and then investigated the underlying neuroprotective mechanisms in glutamate-damaged PC12 cells.

## Material and methods

### Material and reagents

Longistyline C was isolated from the leaves of pigeonpea by our laboratory [18] and the purity is over 95% which was detected by high performance liquid chromatography (HPLC) method.

Dulbecco’s modified Eagle’s medium (DMEM; Gibco, Grand Island, NY), penicillin (100 IU/ml Sigma, St. Louis, MO), streptomycin (10 μg/ml; Sigma), 10% heat-inactivated fetal calf serum (Gibco, Grand Island, NY). PC12 cells were purchased from the Cell Bank of Peking Union Medical College (Beijing, China). Methyl thiazolyl tetrazolium (MTT), dimethylsulfoxide (DMSO) and glutamate were purchased from Sigma-Aldrich Inc. (St. Louis, MO, USA), Fura-2/AM (Beyotime Institute of Biotechnology, Jingsu, China). Antibodies used for western blot analysis, primary antibodies: 1:1000 Anti-GADPH, 1:200 Anti-ERK1/2, 1:200 Anti-β-Actin, 1:100 Anti-GRP78, 1:200 Anti-p-ERk1/2,1:250 Anti-Caspase-12, 1:100 Anti-GADD153, 1:200 Anti-XBP-1, 1:500 Anti-Caspase-9, 1:1000 Anti-NMDAR/NR2B, 1:1000 Anti-CaMKII, 1:1000 Anti-CREB and 1:1000 Anti-p-CREB (Cell signaling Technology, USA); secondary antibodies: 1:1000 Anti-mouse-HRP and 1:1000 Anti-rabbit-HRP (Santa Cruz biotechnology, USA); Nonfat milk powder and bovine serum albumin (Becton, Dickinson and Company, USA). Double distilled water was used in all experiments. All the reagents were of analytical grade.

### Animal

Male ICR mice (17–19 g) were purchased from the Laboratory Animal Institute of the Chinese Academy of Medical Science Center (Beijing, China). The mice were housed in groups of 6 animals per cage under a constant temperature (23°C ±2°C) and humidity (50% ±10%) on a 12/12-h light/dark cycle (lights on from 08:00 to 20:00 hours). Mice had free access to standard chow diet and sterilized drinking water in the SPF animal house. All experimental procedures were conducted under the supervision and approval of the Academy of Experimental Animal Center of the Institute of Medicinal Plant Development and in strict accordance with the NIH Guide for the Care and Use of Laboratory Animals.

### Procedure of antidepressant test in animal test

Mice were randomly separated into 6 groups (n = 12 per group): control (distilled water), vehicle (solvent), positive control (10 mg/kg, paroxetine). The doses of the treated groups were designed as 7.5, 15 and 30 mg/kg of longistyline C according to our preliminary study. All drugs and water were administered by gastric gavages (20 ml/kg) once daily for 11 days between 9:00 a.m. to 11:00 a.m. Behavioral tests were carried as follows: open field test (8^th^ day), tail suspension test (9^th^ day) and forced-swimming test (10^th^ day).

### Open-field test

Prior to the tail suspension and the forced swim tests, the effect of longistyline C on locomotor activities was evaluated in the open-field paradigm using a computerized video-tracking system, which consists of four metal tanks (30 cm in diameter, 40 cm in height) with a video camera fixed at the top. Experiments were performed in a quiet room, and the apparatus was illuminated by alight source of 120 Lux on the ceiling. After 1 h of longistyline C administration, mice were placed individually in one of the four metal containers and locomotor activity was measured for 10 min. The total distance traveled by mice was recorded to evaluate the locomotor activity. Four mice were tested simultaneously.

### Tail suspension test (TST)

The TST was carried out according to the method of Steru et al. (1985), using a computerized device (developed by the Institute of Medicinal Plant Development, the Chinese Academy of Medical Sciences, jointly with Chinese Astronaut Center, China). The apparatus consisted of eight chambers (18 paratus consisted enabled eight mice to be tested simultaneously. Each mouse was suspended by the tail using adhesive tape to a hook connected to a strain gauge. The strain gauge picked up all movements of the mouse and transmitted these to a central unit which calculated the total duration of immobility for the 6 min test. Immobility was defined as the absence of any limb or body movements. With the exception of those caused by respiration, when the mice hang passively and completely motionless. Animals that climbed their tails during testing were excluded from the analysis.

### Forced swim test (FST)

The FST was carried out on mice according to the method of Porsolt (1977). Briefly, mice were individually placed into a glass cylinder (20 cm in height, 14 cm in diameter) filled with 12 cm high water (25 ± 1°C). The total duration of immobility (seconds) was measured during the last 4 min of a single 6 min test session. Mice were considered immobile when they made no attempts to escape except for the movements necessary to keep their heads above the water. The definition of immobility was the absence of all movements with the exception of motions required to maintain the animal’s head above the water. The results are expressed as the time spent immobile during the last 4 min of the 6 min session.

### Cell culture and treatment

PC12 cells were saved in DMEM supplemented with streptomycin (100 μg/mL), penicillin (100 U/mL), 5% horse serum and 5% fetal bovine serum at 37°C in humidified atmosphere of 95% air and 5% CO_2_. For every experiment, cells in the exponential phase of growth were used. Longistyline C was hemolyzeed in dimethyl sulphoxide (DMSO). The final concentration of DMSO was not more than 0.1% (v/v). In all experiments except control groups, glutamate was applied for 24 h after the treatment with longistyline C for 24 h, and cells were washed with PBS twice prior to glutamate treatment. All manipulations were running in 4 duplicates under each treatment for the experiment.

### Cell viability assay

MTT assay was used to measure cell viability. For MTT assay, the cultures were treated with an aliquot (20 μL) of MTT solution (5 mg/mL) in DMEM. The mixture was incubated for 4 h to allow MTT to metabolize to a formazan. Then, DMSO (150 μL) were added to dissolve the formazan. After shaking at room temperature to ensure a homogeneous solution, and the optical densities (OD) were determined at a wavelength of 490 nm by using BIO-RAD Microplate Reader (Model 680, BIO-RAD Laboratories, Hercules, CA, USA). Cell viability was showed as a percentage of the non-treated control.

### Measurement of intracellular calcium level ([Ca^2+^]i)

The concentration of intracellular Ca^2+^ ([Ca^2+^]i) was measured with Fura-2/AM. At the end of treatment, PC12 cells were collected and centrifuged to get the supernatant, then incubated with Fura-2/AM (5 mmol/L, Sigma) at 37°C for 1 h, and centrifuged twice at 1200 g for 4 min. The cells were re-suspended in HEPES buffer solution (137 mol/L NaCl, 5 mmol/L KCl, 1 mmol/L MgCl_2_, 1.5 mmol/L CaCl_2_, 10 mmol/L HEPES and 25 mmol/L D-glucose, PH adjusted to 7.4). The fluorescence intensity was measured by using a microplate reader at the excitation and emission wavelengths of 340 and 500 nm, respectively.

### Measurement of intracellular reactive oxygen species (ROS)

The intracellular ROS level was measured using DCFH-DA [19]. DCFH-DA is a non-fluorescent compound that is enzymatically converted to the strongly fluorescent compound DCF in the presence of ROS. Briefly, PC12 cells were seeded into a 6-well culture plate at a density of 6×10^5^ cells/well. At the end of treatment, the cells were washed with D-Hanks and incubated with DCFH-DA at a final concentration of 10 μM for 30 min at 37°C in darkness. The cells were then washed 3 times with PBS to remove the extracellular DCFH-DA, and the fluorescence intensity of the DCF was measured with a fluorescent microplate reader at an excitation wavelength of 485 nm and an emission wavelength of 538 nm, and the intracellular ROS levels were expressed as percentage of control.

### Measurement of lactate dehydrogenase (LDH) release

The cell damage was identified by the amount of LDH releasing into the incubation medium via the assay kit depending on the manufacturer’s instructions (Nanjing Jiancheng Bioengineering Institute, Nanjing City, China). Briefly, after the drug treatment, the cells in 6-well plate were centrifuged at 1000 g for 4 min, 1 mL culture supernatants were collected from each well; subsequently reaction buffer (3.4 ml) was then added. After mixing at room temperature for 30 min, the release of LDH was measured by a microplate reader (440 nm) and expressed as a percentage (%) of total LDH activity, which was accordded to the equation % LDH released = (LDH activity in the medium/total LDH activity)×100. Each experiment was performed for at least three times.

### Annexin V/PI double staining

Apoptosis rate was determined using the Annexin V/PI assay kit according to the manufacturer’s instruction. The phospholipid phosphatidylserine that accumulates on the extracellular surface can be detected by annexin V. PI (propidium iodide) is a fluorescent dye that binds to the nuclei of dead cells. PC12 cells (1 × 10^5^ cells/well) were cultured in 6-well plates. At the end of the drug treatment, the cells were harvested, washed with cold PBS and incubated in dark with 1× annexin V working solution containing PI (1 μg/mL final concentration) for 20 min at room temperature. Apoptosis rates were determined using a FACS Calibur flow cytometer (BD Biosciences, CA, USA) after the addition of 400 μL of 1× binding buffer.

### Western blot analysis

All protein concentrations were determined by using the Bio-Rad Protein Assay kit (Bio-Rad Laboratories, Berkeley, CA, USA) according to the manufacturer’s protocol. In short, ice-cold modified radio immunoprecipitation (RIPA) buffer was used to collected cells at the end of treatments. After homogenizing and centrifuging the samples at 13000 g for 20 min at 4°C, bradford reagent was added and the absorbance was measured at 595 nm with a microplate reader. The protein concentration was determined with a bovine serum albumin (BSA)basing on the standard curve. And total cell lysates were boiled at 100°C for 5–10 min in 5×sample buffer.

For Western blot analysis, after separating equal amounts of proteins (20 μg) by 8~12% sodium dodecyl sulfate-polyacrylamide gel electrophoresis (SDS-PAGE), resolved proteins were then transferred onto nitrocellulose membranes (0.45μm HATF, Millipore, USA). Then the membranes were blocked with 5% non-fat milk for 3 h and exposed to the appropriate antibodies. The membranes were then incubated using primary antibodies which were diluted in BSA (5%) in a TBST solution overnight at 4°C. Later, the membranes were washed with TBST solution for 3 times and then incubated with the respective horseradish peroxidase-conjugated secondary antibody at room temperature for 1.5hours. Blots were developed with a Super Signal West Femto Trial kit (Thermo Scientific, Rockford, USA) for 5 min, the protein bands were visualized by Molecular Imager (Molecular Imager Chem Doc XRS, Bio-Rad, CA, USA). Optical density of each band was analyzed with Image Lab software (Bio-Rad, CA, USA). Each result was performed for at least three experiments.

### Statistical analysis

Followed by Dunnett’s test to detect any inter-group differences, multiple group comparisons were performed by one-way analysis of variance (ANOVA). Differences were considered to be statistically significant at p < 0.05. Results were expressed as the mean ± standard deviation (SD).

## Results

### Effect of antidepressant of longistyline C on Sub-chronic treated mice

**Fig 2:**
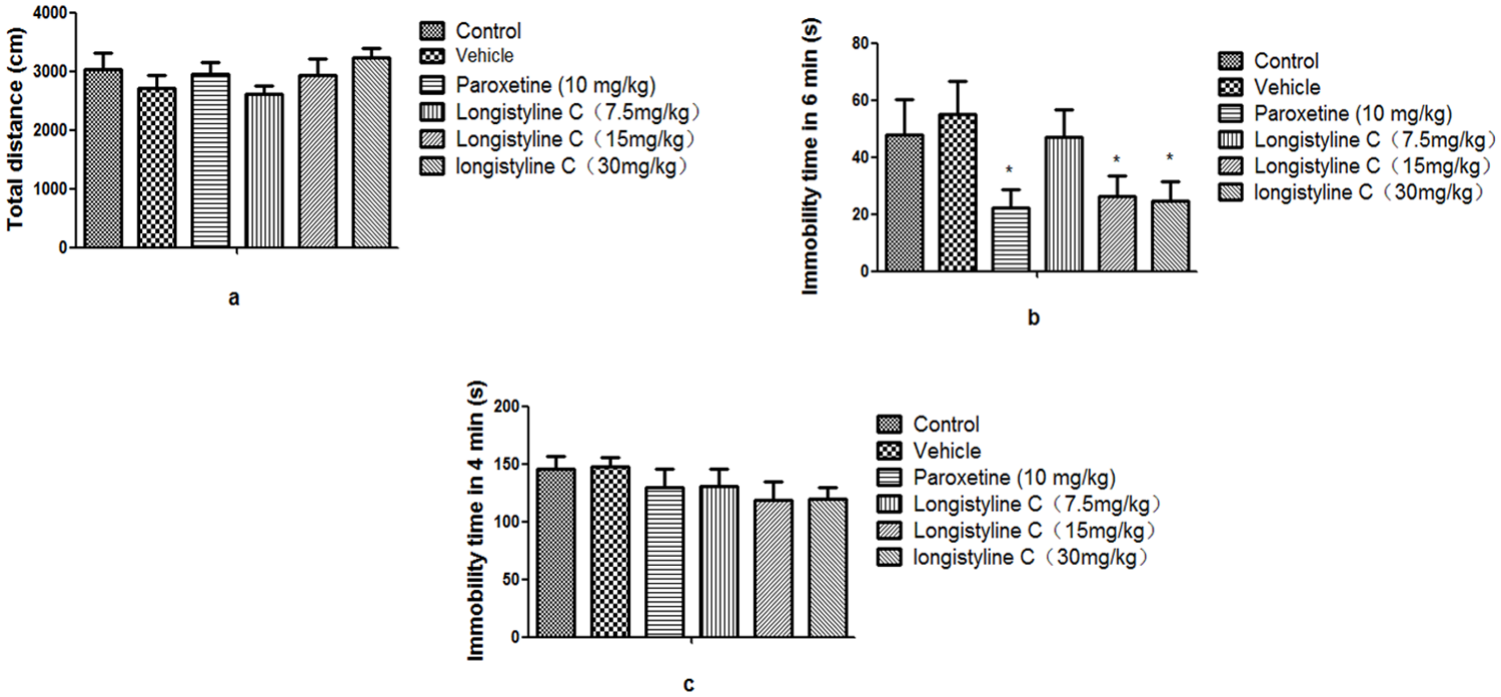
Effect of antidepressant of longistyline C on Sub-chronic treated mice. (a) Effect of longistyline C on the movement distance in the open field test; (b) Effect of longistyline C on the immobility time in the tail suspension test; (c) Effect of longistyline C on the immobility time in the forced swim test Distilled water, solvent, longistyline C (7.5, 15 and 30 mg/kg) and paroxetine (10 mg/kg) were orally administered for 11 days, respectively. Values given are the mean ± SEM (n = 12). For statistical significance, *P < 0.05, **P < 0.01, as compared with the control group.

As shown in Fig 2a, no significant effect of longistyline C (7.5, 15 and 30mg/kg) and the positive control paroxetine (10mg/kg) on the total distance of mice was observed in the open-field test, indicating longistyline C not affecting the mice locomotive activities.

As revealed in Fig 2b, longistyline C (15 and 30 mg/kg) and paroxetine (10 mg/kg) treatment for 10 days significantly reduced immobility time as compared with control mice (p < 0.05). Longistyline C (7.5 mg/kg) showed no effect to reduce immobility time as compared with control mice.

As shown in Fig 2c, three doses of longistyline C and paroxetine (10 mg/kg) treatment for 11 days showed no significantly reducing immobility time in the FST as compared with control mice.

### Effect of longistyline C on glutamate-induced PC12 cells by MTT assay

**Fig 3:**
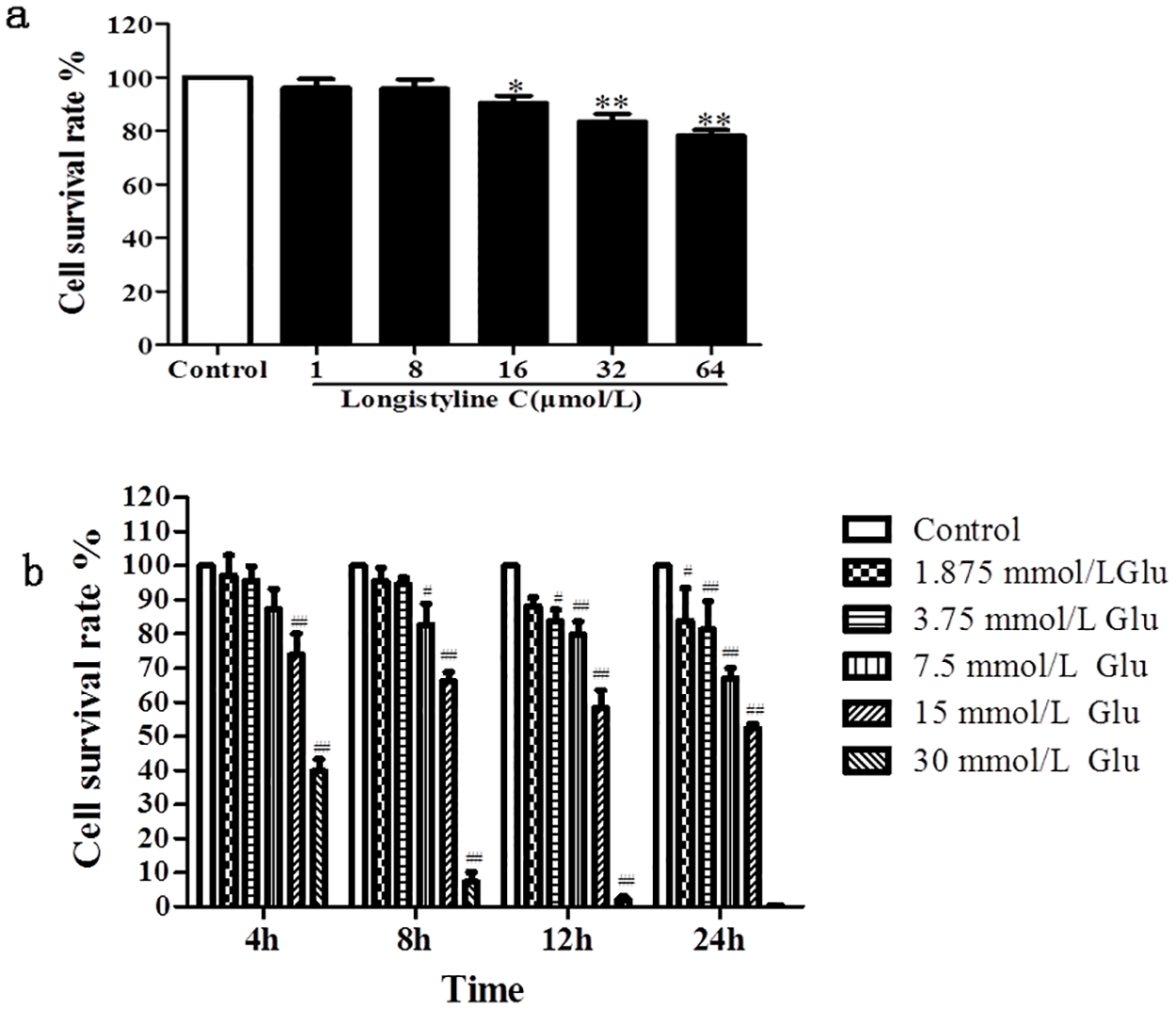
Effects of longistyline C and glutamate at different concentrations on PC12 cells by MTT assay. (a) Effect of longistyline C on PC12 cells; (b) Effect of glutamate on PC12 cells. Each column represents the mean ± SD of four individual experiments (n = 4); *P<0.05 and **P <0.01 as compared with control group. Glu: glutamate.

Firstly, the effects of longistyline C and glutamate at different concentrations on PC12 cells were examined using MTT assay. As shown in Fig 3a and 3b, the significant cytotoxicity of longistyline C was shown when the concentration was beyond 16 μmol/L. Meanwhile, when 15 mmol/L glutamate exposed to PC12 cells for different hours, the cell viability were about 73%, 66%, 58% and 52% at 4 h, 8 h, 12 h and 24h, respectively. Based on these results, the concentrations of longistyline C were chosen 1–16 μmol/L, and the concentration and time of glutamate was selected 15 mmol/L and acting 24 h for the subsequent experiments.

**Fig 4:**
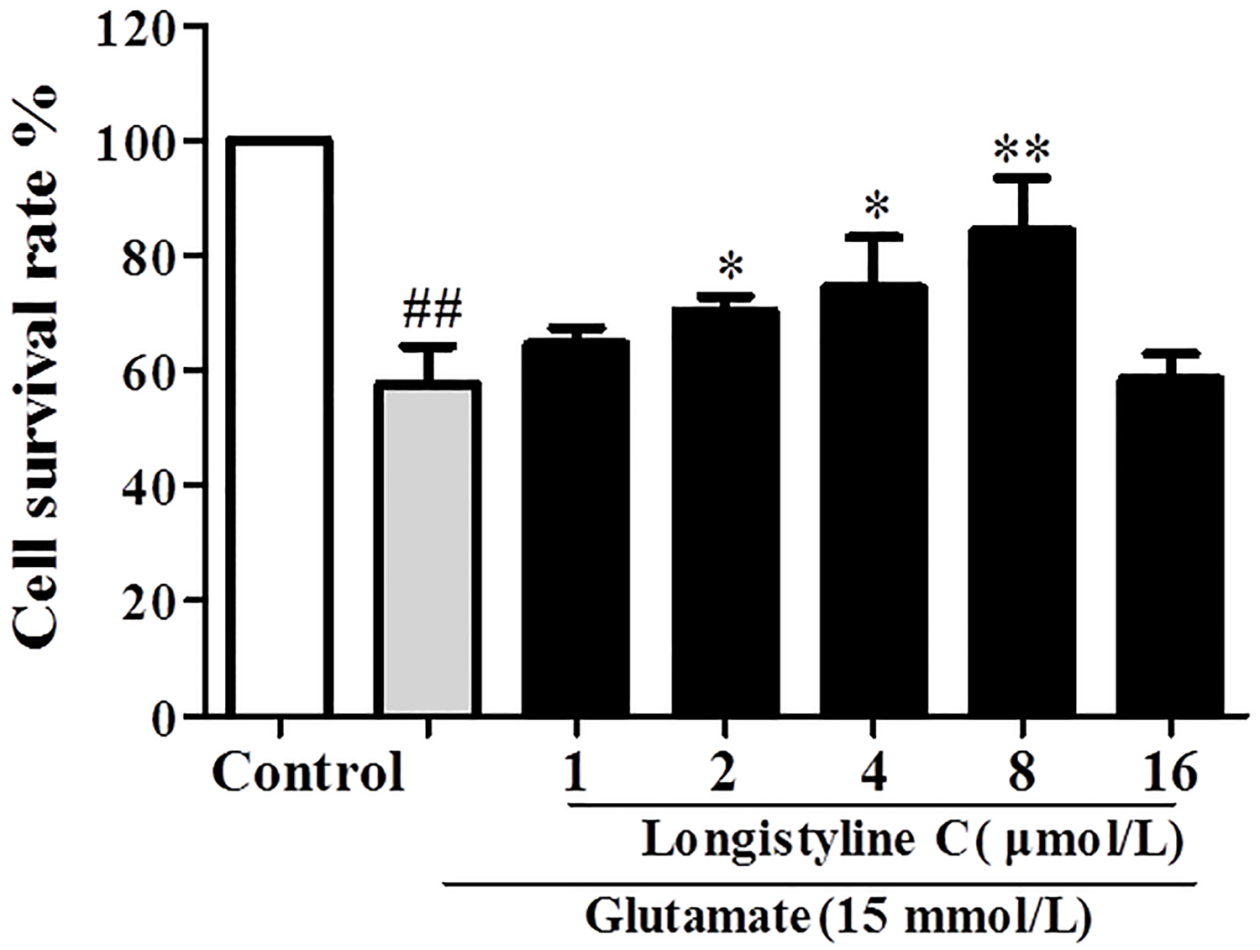
Protective effects of longistyline C on glutamate-induced PC12 cells by MTTassay. Each column represents the mean ± SD of four individual experiments (n = 4) **P<0.01, *P<0.05 compared with glutamate group; ##P<0.01 compared with control group.

Secondly, we detected the protective effects of longistyline C (1–16 μmol/L) on glutamate (15 mmol/L)-induced PC12 cells by MTT assay. As shown in Fig 4, stimulation with glutamate alone resulted in a significant decrease in cell viability as compared with the control group; on the contrary, pretreatment with longistyline C in the presence of glutamate exhibited a significant decrease of glutamate-induced toxicity in a concentration-dependent manner under 2–8 μmol/L of longistyline C. Among the effective concentrations, 8 μmol/L of longistyline C showed the best neuroprotective effect, which increased the cell survival rate from 57.6±5.8% (glutamate-induced group) to 84.4±7.8%. However, the cell apoptosis intensified when longistyline C was 16 μmol/L, and indicated the cytotoxicity of longistyline C. Therefore, 2, 4 and 8 μmol/L were chosen to do the experiments of LDH release and intracellular calcium ([Ca^2+^]i) and ROS measurement, while 8 μmol/L was selected in the investigation of Annexin V/PI double staining and western blot analysis.

### Longistyline C alleviated intracellular calcium concentration ([Ca^2+^]i) in glutamate-induced PC12 cells

**Fig 5:**
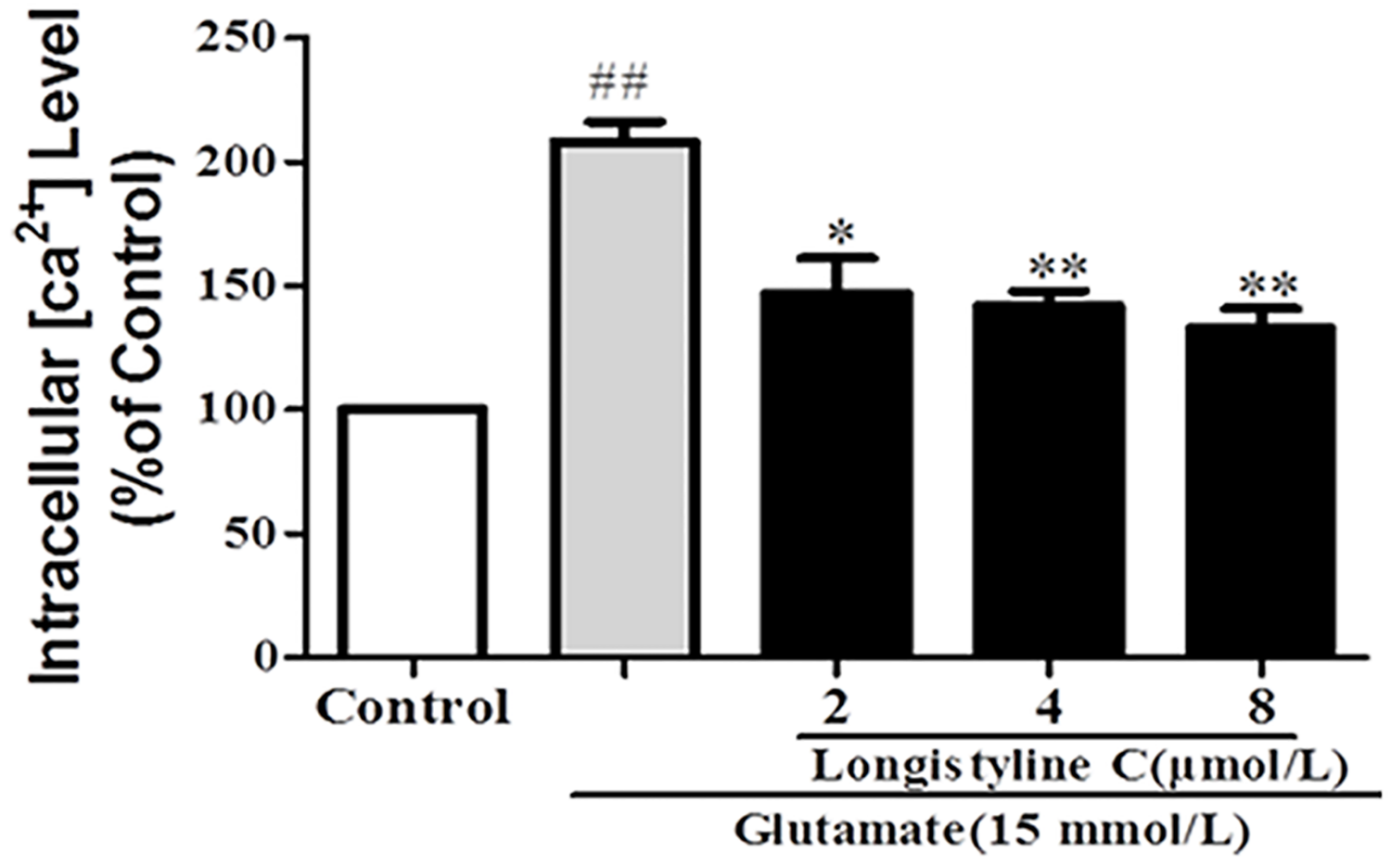
Effect of longistyline C on [Ca2+]i in glutamate-treated PC12 cells. The values given are the mean ± SD (n = 4); **P <0.01, *P<0.05 compared with glutamate group; ## P<0.01 compared with control group.

The [Ca^2+^]i concentration was measured by Fluo-2/AM fluorescence labeling assay. As shown in Fig 5, treatment with glutamate (15 mmol/L) for 24 h, the ratio of fluorescence intensity of [Ca^2+^]i in PC12 cells was significantly increased as compared with the control group (p<0.01) and the concentration of [Ca^2+^]i was 208.3±7.0%, while for the control group it was 100%. However, longistyline C (2, 4 and 8 μmol/L) significantly decreased the fluorescence intensities of [Ca^2+^]i as compared with glutamate-treated group, which were 147.1± 12.2%, 141.9 ± 5.3% and 132.9 ± 6.7%, respectively. The results revealed that longistyline C can significantly attenuate the intracellular Ca^2+^overloading induced by glutamate in PC12 cells.

### Longistyline C alleviated glutamate-induced ROS contents in PC12 cells

**Fig 6:**
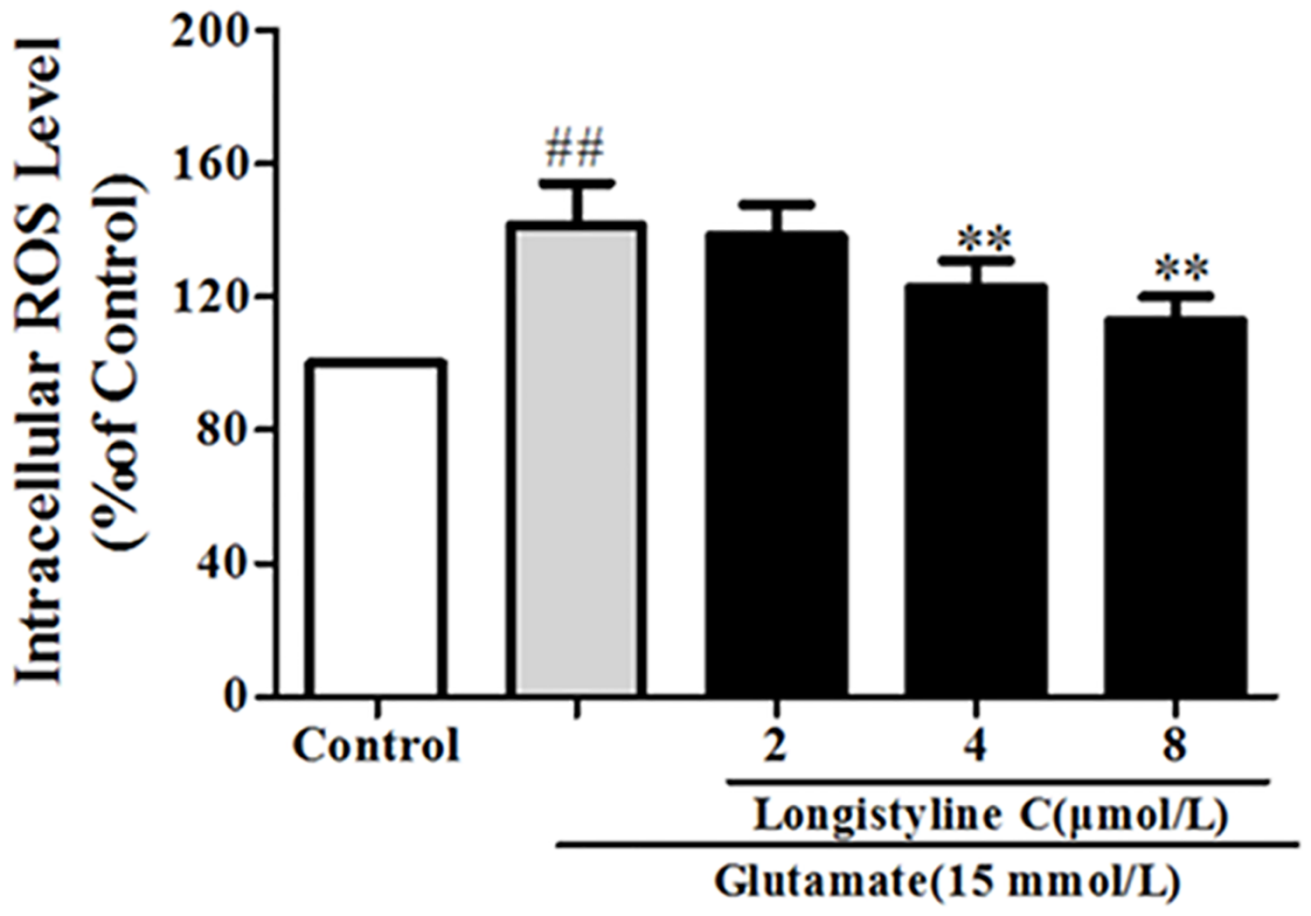
Effect of longistyline C on ROS the levels in glutamate-induced PC12 cells. Each column represents the mean ± SD (n = 4); **P <0.01, compared with glutamate group; ## P<0.01 compared with control group.

As shown in Fig 6, treatment with glutamate could cause a significant increase in the intracellular ROS levels as compared with the control group (P<0.01), and the ROS level was 144.0 ± 9.3% of the control group. However, the levels were significantly reduced when treated with 4, 8 μmol/L longistyline C for 24 h as compared with the glutamate groups, the contents of which were 122.8 ± 6.9% and 112.7 ± 6.5%, respectively.

### Longistyline C rescued glutamate-induced apoptosis in PC12 cells

**Fig 7:**
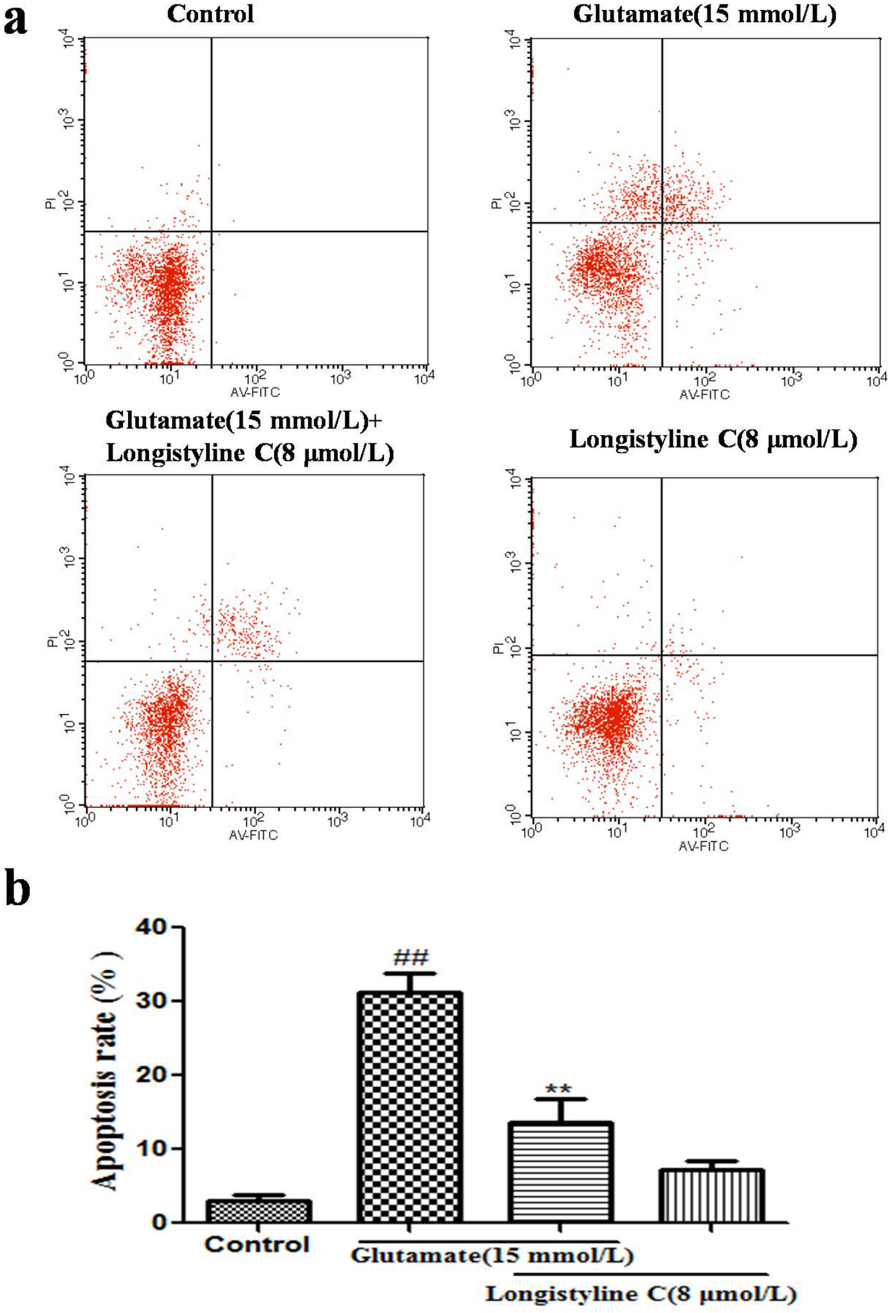
Effects of longistyline C on the cell survival in glutamate treated PC12 cells by Annexin V—PI double staining kits through flow cytometry. Results were expressed as mean ± SD (n = 3). ##P<0.01 as compared with the control group; **P<0.01 as compared with glutamate group.

As shown in Fig 7a and 7b, annexin+/PI and annexin+/PI+ were substantially increased in the glutamate-treated cells. This result suggested that the apoptosis rate of PC12 cells was significantly increased by glutamate challenge and the cell apoptosis rate increased to 30.91% of the control group. However, these changes were markedly reversed by longistyline C pre-incubation. These results suggested that longistyline C was capable of rescuing PC12 cells from glutamate-induced apoptosis.

### Longistyline C alleviated LDH leakage

**Fig 8:**
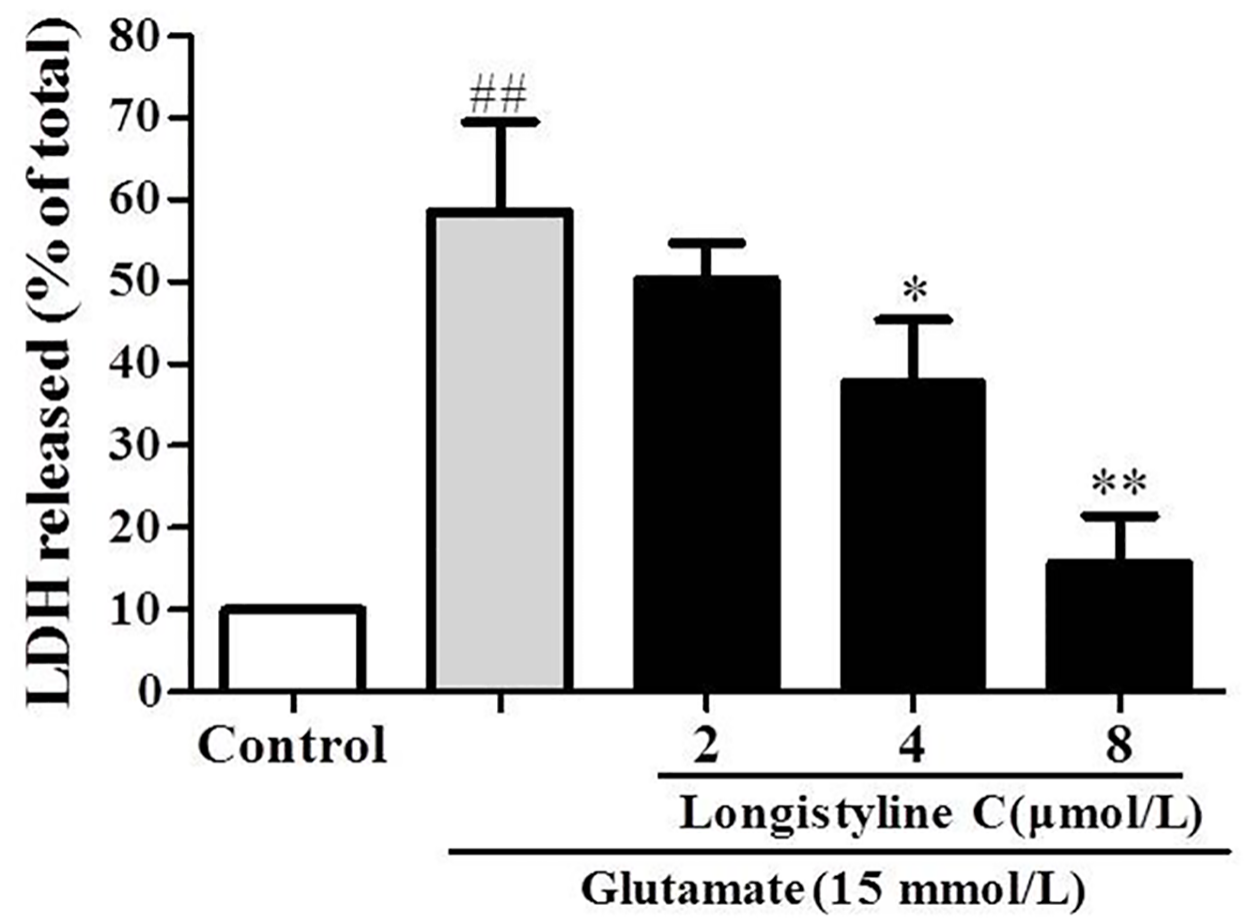
Effect of longistyline C on LDH leakage in glutamate-treated PC12 cells. The values given are the mean ± SD (n = 3); **P <0.01, *P<0.05 compared with glutamate only group; ## P<0.01 compared with control group.

As depicted in Fig 8, the level of LDH release increased to 58.6±9.5% after exposure to glutamate. However, pretreatment with longistyline C resulted in a significant decrease at the concentration of 2, 4 and 8 μmol/L as compared with the glutamate group (P>0.05, P<0.05 and P<0.01, respectively) and the LDH release were 50.2± 3.9%, 37.7 ± 6.7% and 15.7 ± 4.9%.

### Longistyline C downregulated NR2B and CaMKII expressions and upregulated p-ERK1/2 expression in glutamate pretreated PC12 cells

**Fig 9:**
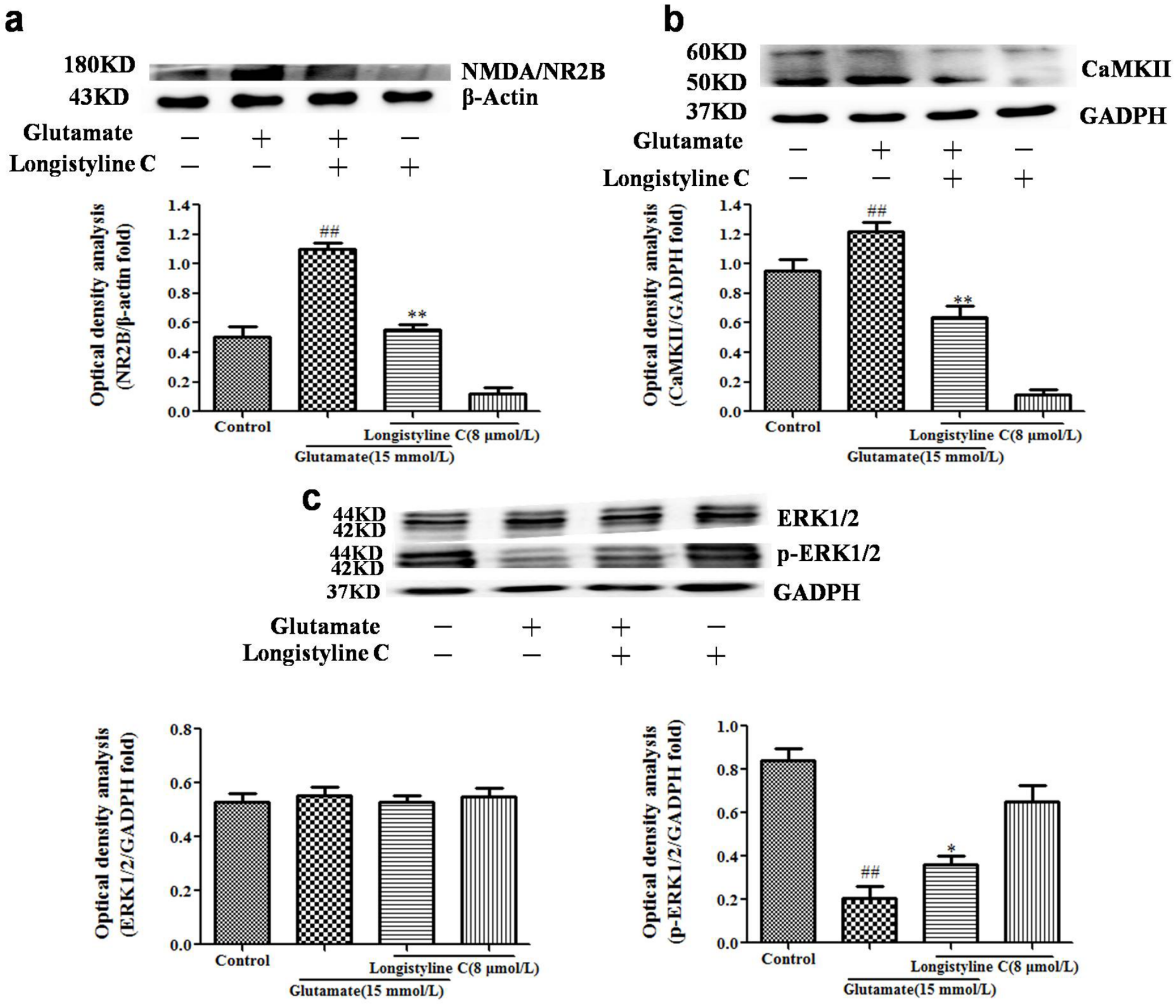
Effects of longistyline C on expressions of NR2B, CaMKII, ERK and p-ERK proteins. The data are presented as the means ± SD (n = 3). Densitometric analyses of protein bands were normalized to a loading control GAPDH (or β-actin). ##P<0.01 as compared with the control group; *P<0.05, **P<0.01 as compared with the glutamate group. All experiments included in control, glutamate, longistyline C and glutamate+longistyline C groups using GAPDH (or β-actin) as the loading control. (a) Expression of NR2B; (b) Expression of CaMKII; (c) Expression of ERK1/2 and p-ERK1/2. Blots in Fig 9a, b, c are cropped for clarity and conciseness.

The expression of NR2B and CaMKII were drastically elevated in the glutamate treated group as compared with the control group (p<0.01), but down-regulated by longistyline C treatment (P<0.01) (Fig 9a and 9b). Meanwhile, after glutamate exposure, the expression of phosphorylated ERK1/2 (p-ERK1/2) was markedly decreased (P<0.01) (Fig 9c), but significantly elevated with longistyline C treatment (P<0.05), while the expressions of ERK1/2 make no difference in all groups. In addition, treatment with longistyline C alone could slightly down-regulate NR2B and CaMKII and up-regulate the phosphorylated ERK1/2.

### Longistyline C promotedp-CREB activation

**Fig 10:**
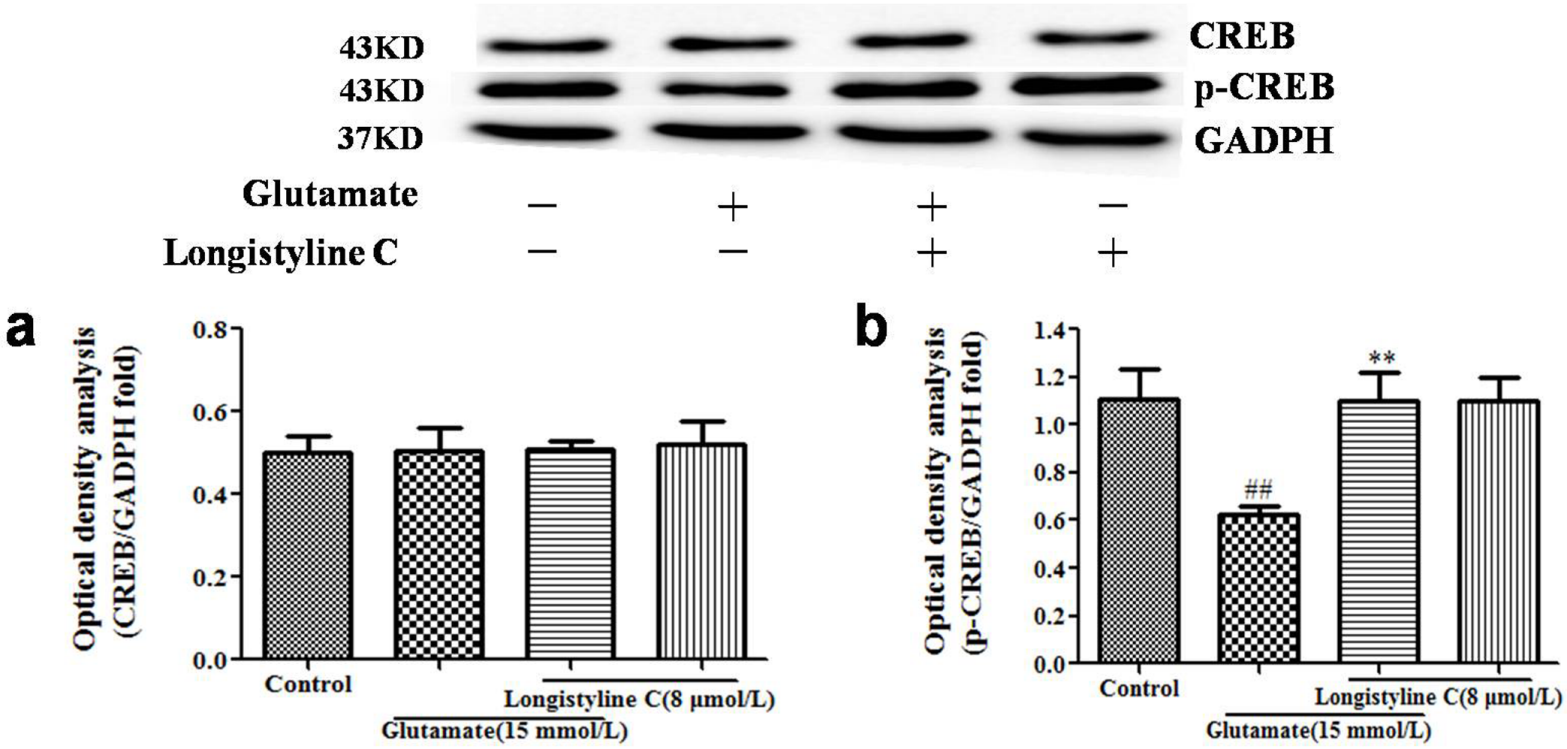
Effects of longistyline C on expressions of p-CREB and CREB. The data are presented as the means ± SD (n = 3). Densitometric analyses of protein bands were standardized to a loading control GAPDH. ##P<0.01 as compared with the control group; **P<0.01 as compared with the glutamate group. All experiments included in control, glutamate, longistyline C and glutamate+longistyline C groups using GAPDH as the loading control. (a) Expression of CREB; (b) Expression of p-CREB. Blots in Fig 10 is cropped for clarity and conciseness.

To identify downstream effects of ERK1/2 pathway, we measured the ERK1/2 downstream targets p-CREB and CREB. Glutamate exposure resulted in a significant decrease of p-CREB when compared to the control group (P<0.01). However, longistyline C prevented this reduction relative to the glutamate group (P<0.01) (Fig 10), while there were no effects on CREB protein in all groups.

### Longistyline C alleviated glutamate-induced ER stress in PC12 cells

**Fig 11:**
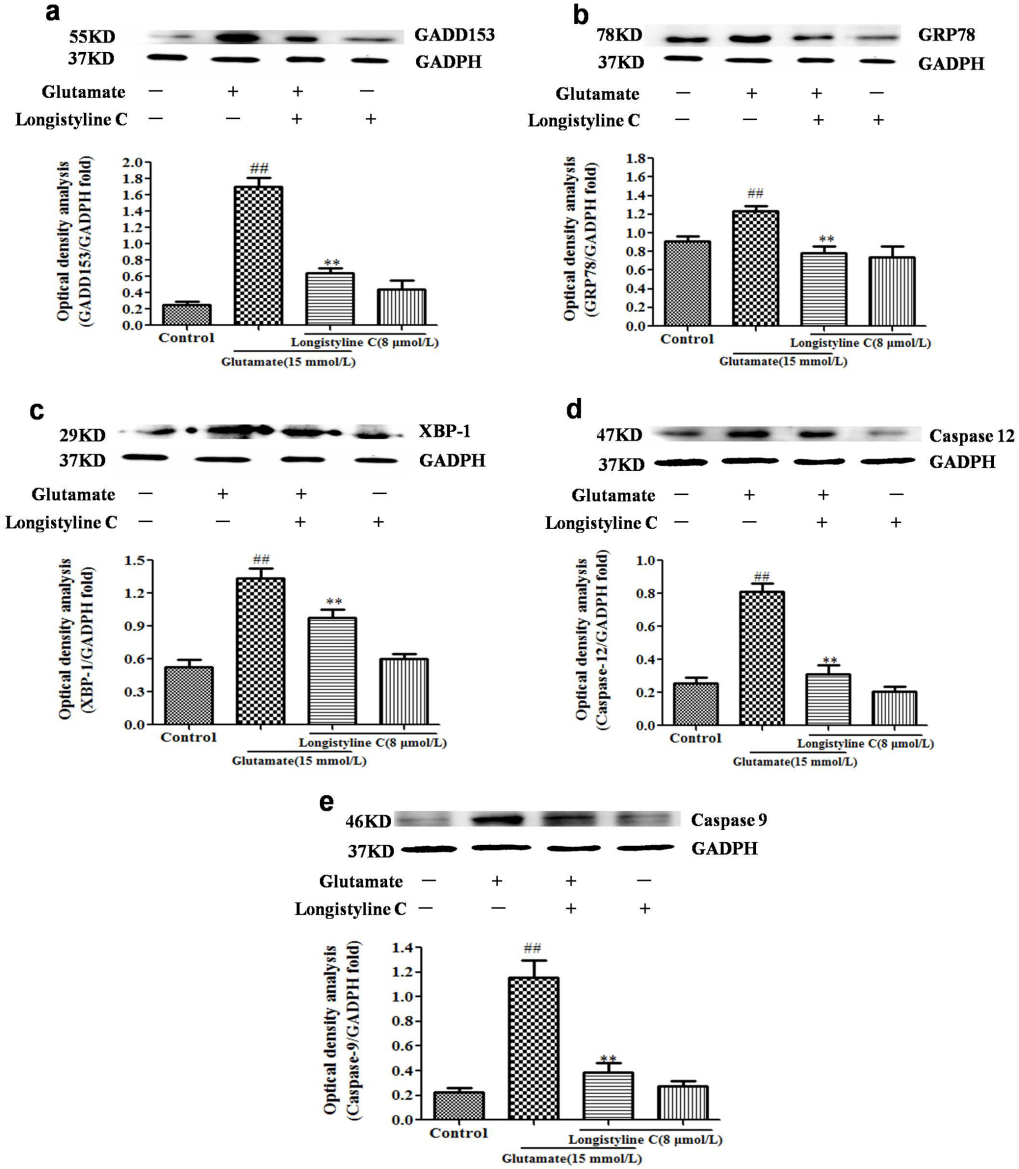
Effects of longistyline C on GADD153, GRP78, XBP-1, caspase-9 and caspase-12 expressions. The data are presented as the means ± SD (n = 3). Densitometric analyses of protein bands were standardized to a loading control GAPDH. ##P<0.01 as compared with the control group; *P<0.05 and **P<0.01 as compared with the glutamate group. All experiments included in control, glutamate, longistline C, and glutamate+longistyline C groups using GAPDH as the loading control. (a) Expression of GADD153; (b) Expression of GRP78; (c) Expression of XBP-1; (d) Expression of caspase-12; (e) Expression of caspase-9. Blots in Fig 11a, b, c, d, e are cropped for clarity and conciseness.

As shown in Fig 11, the glutamate treated PC12 cells were found to have increased protein expressions of GRP78, CHOP/GADD153 and XBP-1, as well as ER-specific proteins as caspase-12 and caspase-9 in comparison to control group, and which were significantly blunted by longistyline C treatment in glutamate treated PC12 cells. Further, treatment with longistyline C alone didn’t have much influence on the expressions of the above proteins when compared with control group.

## Discussion

FST and TST are based on the fact that animals subjected to short-term, inescapable stress will develop an immobile posture. This immobility, referred to as behavioral despair in animals, is claimed to reproduce a condition similar to human depression [19]. Our present study indicates that longistyline C (15 and 30mg/kg) significantly reduces the duration of immobility in tail suspension test, which displayed the similar effect to the positive control paroxetine. There was no change in the locomotor activity of animals treated with longistyline C, implying that longistyline C could have the antidepressant-like effect without affecting locomotor activity.

Based on the animal tests, we investigated the neuroprotective effects of longistyline C against glutamate-induced neurotoxicity in PC12 cells. The result demonstrated that longistyline C presents marked neuroprotective effects, which was confirmed by MTT, LDH assays, in parallel with apoptosis analysis obtained by flow cytometer experiments. Meanwhile, we also proved that this neuroprotection was connected with regulating of NMDAR/NR2B-ERK1/2 pathway and suppressing ER stress.

NMDARs are gated by the neurotransmitter glutamate and play central roles in a number of physiological processes [20]. NMDARs consist of three different subtypes: NR1, NR2 and NR3. NR1 (also called GluN1)has eight different subunits, NR2 has four subunits (A—D, also called GluN2A—D) and NR3 has two subunits (A and B, also called GluN3A and GluN3B). Previous studies reported that NR2A and NR2B play different roles in the CNS [21, 22] and NR2B-NMDARs were found to be capable of promoting both survival and death signaling [23]. In pathophysiological conditions, such as ischaemia and/or hypoxia, or neurodegeneration, NR2B subunits trigger cell destructive pathways. The persistent activation of extra synaptic NR2B is responsible for excitotoxicity [24, 25]. The mitogen-activated protein kinase phosphatase (MAPK) signaling cascade transduces extracellular information to the nucleus and regulates cell growth and differentiation [26–28]. One of the best-characterized MAPK pathways involves extracellular signal-regulated kinase 1 and 2 (ERK1/2). Studies have demonstrated ERK signalling cascade is a crucial pathway in mediating NMDAR dependent neuronal plasticity and survival [29, 30]. Normal expressed NMDAR-dependent signalling activates the ERK1/2 cascade with pro-survival consequences including CREB and BDNF activation [31]. In current study, we first investigated the effect of longistyline C on NR2B subunit, then introduced ERK signaling cascade and measured the ERK1/2 downstream targets p-CREB. The results revealed that longistyline C treatment could decrease the expression of up-regulated NR2B subunits and increase the down-regulated expressions of p-ERK1/2.

CaMKII is a multifunctional, calcium-activated kinase (1 and 2), whose α and β isoforms are particularly abundant in brain cytosol and in postsynaptic densities [32]. The activated CaMKII can bind to NR2B with high affinity and the association between active CaMKII and NR2B is required for the maintenance of synaptic plasticity [33]. In pathophysiological conditions, the sustained increasing concentration of glutamate induces the excessive expression of NR2B, resulting in uncontrolled Ca^2+^ influx and activation of CaMKII and its downstream effectors, which triggers excitotoxicity [34, 35]. In present study, we observed excessive and prolonged glutamate stimulated PC12 cells, resulting over expressions of NMDAR/NR2B subunit and CaMKII protein, as well as dow-regulation the expressions of p-ERK and p-CREB. Nevertheless, pretreatment with longistyline C could partially reverse the four protein expressions (Fig 9).

Interestingly, we noticed that treatment with longistyline C alone could down-regulate expressions of NR2B and CaMKII, and upregulate expressions of p-ERK1/2 and p-CREB. Longistyline C, a stilbene from Cajanuscajan, is structurally related to the estradiol, is considered as phytoestrogen, and has estrogen-like pharmacological effects. Therefore, it is speculated that longistyline C protected PC12 cells against glutamate-induced injury likely via estrogen-receptor relative pathways. A detailed investigation is still needed to elucidate this puzzle.

As a major storage organelle for calcium in cell, ER takes charge of synthesis and folding of proteins [36]. Compelling evidences have led to the fact that excessive influx of extracellular Ca^2+^ and massive accumulation of ROS could cause the accumulation of unfolded or misfolded proteins in the ER and further evoke the dysfunction of unfold protein response (UPR), resulting in the onset of ER stress and triggering cell apoptosis [37]. The results revealed that the neuroprotective effects of longistyline C in glutamate-induced PC12 cells might be mediated, at least partly, by inhibition of intracellular [Ca^2+^] (Fig 5) and ROS concentrations (Fig 6). Under ER stress, the major ER-localized chaperones: Glucose-regulated protein 78 (GRP78) which is one of the most abundant glycoprotein in the ER is induced, and activation of GRPs is demonstrated to regulate toxicants or stimulus induced apoptotic pathways [38]. In addition, induction of C/EBP homologous protein (CHOP), known as growth arrest and DNA damage inducible gene 153 (GADD153) also plays a crucial role in underlying ER stress induced apoptosis [39]. The X box-binding protein-1 (XBP-1) which can be a molecular to bind to promoters of several genes involved in UPR [40], responsible for restoring the homeostatic circuits in cells. The present study revealed that PC12 cells exposed to glutamate had significantly increased the expression levels of ER-related chaperones, including GRP 78, CHOP and XBP-1. However, pretreatment with longistyline C could significantly attenuate their expression levels (Fig 11). Beside the ER chaperone proteins, ER-specific caspase-12 protein which belongs to the caspase protein family has been shown to subsequently activate caspase-9 and initiate ER stress-induced apoptosis [41, 42]. The results indicated pretreatment with longistyline C could reverse the increase of the caspase-12 and caspase-9 expressions in glutamate-induced PC12 cells (Fig 11). Based on these results, it is convinced that longistyline C could relieve UPR during ER stress which is triggered by prolonged glutamate insult.

## Conclusions

Longistyline C showed antidepressant effect in tail suspension test, and exerted a neuroprotective effect against glutamate-induced injury in PC12 cells, which the underlying molecular mechanism is most possibly through regulating NMDAR/NR2B-ERK1/2 related signaling pathway, with restoring ER functions. Our results will provide reference for the development of new antidepressants.

## Supporting information

S1 File The data of longistyline C. (PDF)

S2 File The effects of antidepressant on Sub-chronic treated mice treated with different concentrations of longistyline C. (PDF)

S3 File The cell survival rate in PC12 cells treated with longistyline C and glutamate at different concentrations and at different time periods of culture. (PDF)

S4 File The cell survival rate in glutamate-induced PC12 cells treated with different concentrations of longistyline C. (PDF)

S5 File The intracellular Ca^2+^ level in glutamate-induced PC12 cells treated with different concentrations of longistyline C. (PDF)

S6 File The intracellular ROS level in glutamate-induced PC12 cells treated with different concentrations of longistyline C. (PDF)

S7 File The apoptosis rate in glutamate-induced PC12 cells treated with longistyline C (8μM). (PDF)

S8 File The LDH leakage in glutamate-induced PC12 cells treated with different concentrations of longistyline C (8μM). (PDF)

S9 File The expressions of NR2B, CaMKII, ERK and p-ERK proteins in glutamate-induced PC12 cells treated with different concentrations of longistyline C (8μM). (PDF)

S10 File The expressions of p-CREB and CREB proteins in glutamate-induced PC12 cells treated with different concentrations of longistyline C (8μM). (PDF)

S11 File The expressions of GADD153, GRP78, XBP-1, caspase-9 and caspase-12 in glutamate-induced PC12 cells treated with different concentrations of longistyline C (8μM). https://figshare.com/s/d99d1459759757174a66. DOI: 10.6084/m9.figshare.5331466.
(PDF)
