# Supplementary material for: Longistyline C acts antidepressant in vivo and neuroprotection in vitro against glutamate-induced cytotoxicity by regulating NMDAR/NR2B-ERK pathway in PC12 cells
Source: PLoS One. 2017 Sep 5;12(9):e0183702. doi: 10.1371/journal.pone.0183702 (PMC5584824; doi:10.1371/journal.pone.0183702)
Supplement: S1 File — (PDF) [file pone.0183702.s001.pdf]

The data of longistyline C

$C_{20}H_{22}O_2$:ESI-MS *m*/*z*: 293 [M-H]$^-$, ^{1}H-NMR (600 MHz, CD_3OD) δ: 7.51 (2H, d, *J* = 8.4 Hz, H-2’, 6’), 7.32 (2H, t, *J* = 7.2 Hz, H-3’, 5’), 7.22 (1H, t, *J* = 7.2 Hz, H-4), 7.05 (2H, d, *J* = 3.0 Hz, H-7, 8), 6.65 (1H, d, *J* = 1.2 Hz, H-4), 6.63 (1H, d, *J* = 1.2 Hz, H-6), 5.12 (1H, t, *J* = 7.2 Hz, H-2″), 3.83 (3H, s, -OCH3), 3.29 (2H, d, *J* = 7.2 Hz, H-1″), 1.76 (3H, s, H-4″), 1.65 (3H, s, H-5″); ^{13}C-NMR (150 MHz, CD_3OD) δ: 160.2 (C-3), 157.1 (C-5), 139.2 (C-1’), 137.6 (C-1), 131.1 (C-3″), 130.4 (C-7), 129.9 (C-3’, 5’), 128.8 (C-8), 128.6 (C-4’), 127.6 (C-2, 6’), 124.6 (C-2″), 117.9 (C-2), 107.6 (C-6), 102.1 (C-4), 55.8 (3-OCH_3), 25.9 (C-1″), 23.1 (C-4″), 18.1 (C-5″)

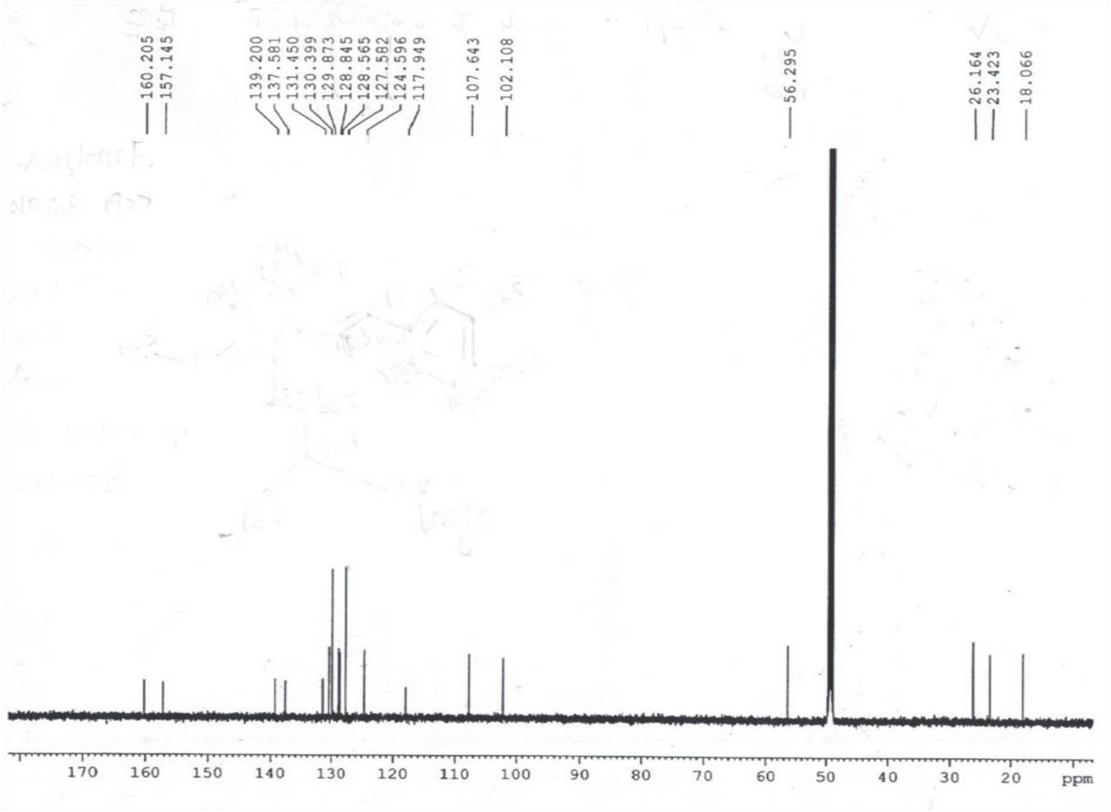

^{13}C-NMR

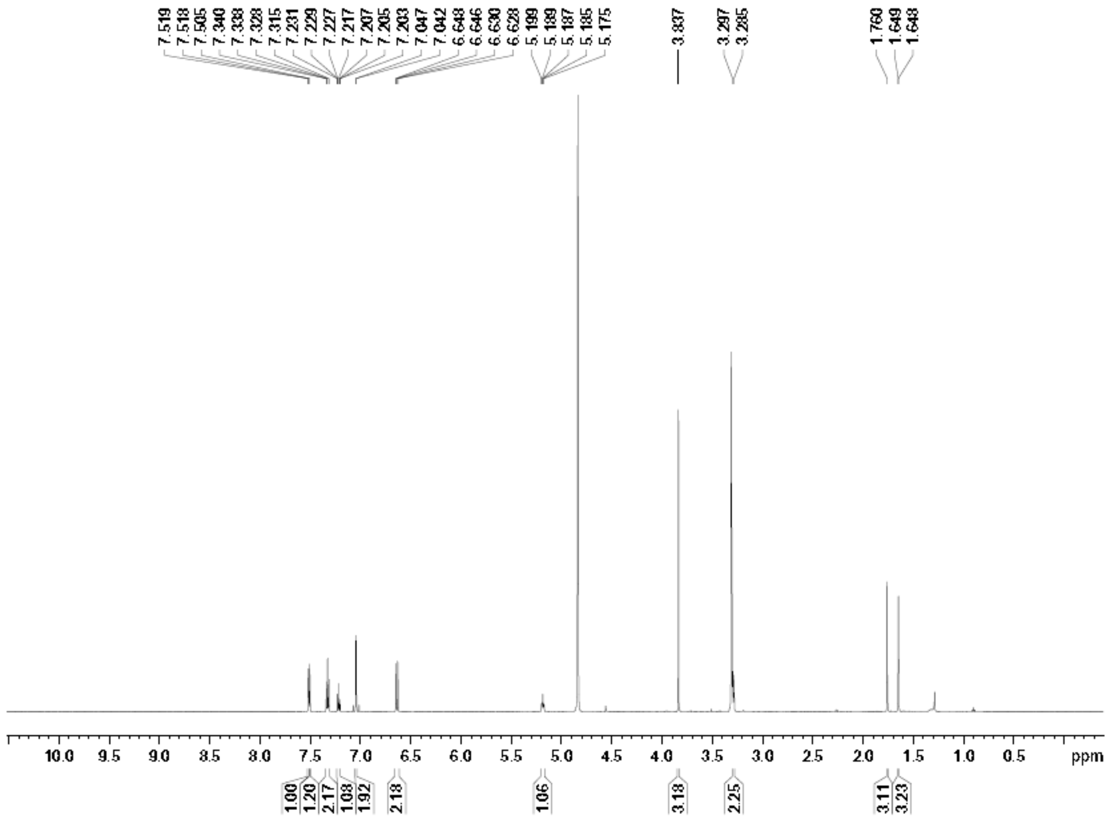

^{1}H-NMR

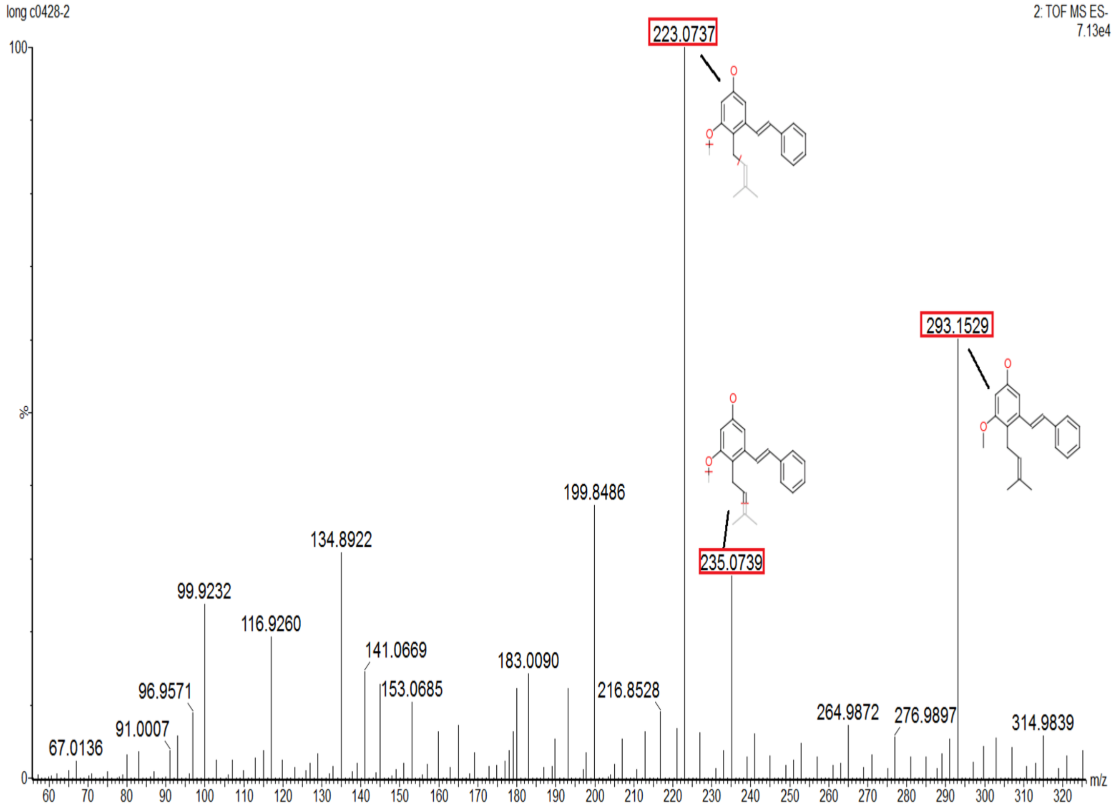

The secondary fragments of longistyline C
