# Supplementary material for: Longistyline C acts antidepressant in vivo and neuroprotection in vitro against glutamate-induced cytotoxicity by regulating NMDAR/NR2B-ERK pathway in PC12 cells
Source: PLoS One. 2017 Sep 5;12(9):e0183702. doi: 10.1371/journal.pone.0183702 (PMC5584824; doi:10.1371/journal.pone.0183702)
Supplement: S2 File — (PDF) [file pone.0183702.s002.pdf]

SUPPORTING INFORMATION

fig.2a

	Mg/kg	N	Total distance（cm）
control	/	12	3023.37±281.98
vehicle	/	12	2722.72±212.45
paroxetine	10	12	2955.44±206.95
longistyline C	7.5	12	2617.31±144.60
longistyline C	15	12	2925.31±295.25
longistyline C	30	12	3226.35±171.74

fig.2b

	Mg/kg	N	Immobility time in 6min (s)	P
control	/	12	47.96±12.17	0.616
vehicle	/	12	55.14±11.57	/
paroxetine	10	11	22.13±6.57*	0.026
longistyline C	7.5	11	47.16±9.41	0.586
longistyline C	15	12	26.11±7.50*	0.021
longistyline C	30	12	24.50±6.90*	0.035

Table C（fig.2c）

	Mg/kg	N	Immobility time in 4 min (s)	P
control	/	12	145.75±11.40	0.916
vehicle	/	12	147.63±8.49	/
paroxetine	10	12	129.70±16.45	0.316
longistyline C	7.5	12	130.86±14.78	0.348
longistyline C	15	12	118.65±15.86	0.106
longistyline C	30	12	119.94±10.04	0.122
