# Supplementary material for: Longistyline C acts antidepressant in vivo and neuroprotection in vitro against glutamate-induced cytotoxicity by regulating NMDAR/NR2B-ERK pathway in PC12 cells
Source: PLoS One. 2017 Sep 5;12(9):e0183702. doi: 10.1371/journal.pone.0183702 (PMC5584824; doi:10.1371/journal.pone.0183702)
Supplement: S3 File — (PDF) [file pone.0183702.s003.pdf]

SUPPORTING INFORMATION

fig.3a

Control	1 µmol/L	8 µmol/L	16 µmol/L	32 µmol/L	64 µmol/L
100.00	92.64	97.62	89.90	82.35	78.76
100.00	99.80	99.78	87.09	87.58	76.10
100.00	97.90	93.12	93.12	83.14	80.92
100.00	94.17	92.59	91.82	80.56	77.31

fig.3b

Time	Control				1.875mmol				3.75mmol				7.5mmol				15mmol				30mmol			
4h	100.00	100.00	100.00	100.00	95.58	94.23	105.93	92.85	89.36	96.82	97.30	99.17	83.39	95.46	88.07	82.05	82.14	74.67	67.47	71.22	36.99	37.57	44.62	39.87
8h	100.00	100.00	100.00	100.00	99.90	90.24	95.03	96.68	96.77	92.91	92.97	95.74	87.47	76.30	78.60	88.58	68.88	63.21	64.32	68.00	10.15	9.19	4.20	5.26
12h	100.00	100.00	100.00	100.00	87.63	86.63	86.33	91.95	87.32	79.63	82.14	85.74	79.11	80.68	75.31	84.42	51.64	56.88	63.17	61.58	3.26	2.19	1.20	1.21
24h	100.00	100.00	100.00	100.00	89.78	73.25	78.75	93.79	84.34	89.62	81.36	69.63	68.96	63.39	65.80	70.05	51.13	52.12	51.30	54.47	0.15	0.19	0.25	0.05
