# Supplementary material for: Longistyline C acts antidepressant in vivo and neuroprotection in vitro against glutamate-induced cytotoxicity by regulating NMDAR/NR2B-ERK pathway in PC12 cells
Source: PLoS One. 2017 Sep 5;12(9):e0183702. doi: 10.1371/journal.pone.0183702 (PMC5584824; doi:10.1371/journal.pone.0183702)
Supplement: S4 File — (PDF) [file pone.0183702.s004.pdf]

SUPPORTING INFORMATION

fig.4

Control		1 μmol/L	2 μg/mL	4 μg/mL	8 μg/mL	16 μg/mL
100.00	64.16	60.71	73.21	76.21	75.46	62.60
100.00	61.79	67.09	70.78	80.99	78.20	60.42
100.00	55.07	66.12	67.17	78.86	94.65	58.80
100.00	49.45	64.74	70.24	61.64	89.14	52.44
