# Supplementary material for: Longistyline C acts antidepressant in vivo and neuroprotection in vitro against glutamate-induced cytotoxicity by regulating NMDAR/NR2B-ERK pathway in PC12 cells
Source: PLoS One. 2017 Sep 5;12(9):e0183702. doi: 10.1371/journal.pone.0183702 (PMC5584824; doi:10.1371/journal.pone.0183702)
Supplement: S5 File — (PDF) [file pone.0183702.s005.pdf]

SUPPORTING INFORMATION

fig.5

Control		2 μmol/L	4μmol/L	8μmol/L
100.00	218.0328	136.0656	139.3443	132.7869
100.00	201.6949	144.0678	150.8475	127.2881
100.00	211.8644	140.678	140.678	143.8983
100.00	201.5385	167.6923	136.9231	127.6923
