# Supplementary material for: Longistyline C acts antidepressant in vivo and neuroprotection in vitro against glutamate-induced cytotoxicity by regulating NMDAR/NR2B-ERK pathway in PC12 cells
Source: PLoS One. 2017 Sep 5;12(9):e0183702. doi: 10.1371/journal.pone.0183702 (PMC5584824; doi:10.1371/journal.pone.0183702)
Supplement: S6 File — (PDF) [file pone.0183702.s006.pdf]

SUPPORTING INFORMATION

fig.6

Control		2 μmol/L	4μmol/L	8μmol/L
100.00	137.53	144.65	127.27	117.41
100.00	137.69	139.46	117.37	102.05
100.00	130.79	125.01	114.92	112.92
100.00	160.08	144.54	131.82	118.38
