# Supplementary material for: Longistyline C acts antidepressant in vivo and neuroprotection in vitro against glutamate-induced cytotoxicity by regulating NMDAR/NR2B-ERK pathway in PC12 cells
Source: PLoS One. 2017 Sep 5;12(9):e0183702. doi: 10.1371/journal.pone.0183702 (PMC5584824; doi:10.1371/journal.pone.0183702)
Supplement: S7 File — (PDF) [file pone.0183702.s007.pdf]

SUPPORTING INFORMATION

fig.7a

Quad%				
Gated	Control	GLU	GLU+LONC	LONC
UL	2.63	14.47	3.16	2.67
UR	0.10	10.97	10.90	1.07
LL	97.05	69.08	84.32	92.63
LR	0.22	5.48	1.62	3.63

fig.7b

Apoptosis%			
Control	GLU	GLU+LONC	LONC
3.00	31.00	15.68	8.00
3.20	34.00	12.00	7.30
1.90	32.00	10.00	8.30
4.00	28.00	17.00	5.60
