# Supplementary material for: Longistyline C acts antidepressant in vivo and neuroprotection in vitro against glutamate-induced cytotoxicity by regulating NMDAR/NR2B-ERK pathway in PC12 cells
Source: PLoS One. 2017 Sep 5;12(9):e0183702. doi: 10.1371/journal.pone.0183702 (PMC5584824; doi:10.1371/journal.pone.0183702)
Supplement: S8 File — (PDF) [file pone.0183702.s008.pdf]

SUPPORTING INFORMATION

fig.8

Control		2 μmol/L	4 μmol/L	8 μmol/L
10.00	46.25	50.00	33.75	12.50
10.00	72.00	55.00	38.00	24.00
10.00	54.29	44.29	30.71	11.43
10.00	61.67	51.67	48.33	15.00
