# Supplementary material for: Longistyline C acts antidepressant in vivo and neuroprotection in vitro against glutamate-induced cytotoxicity by regulating NMDAR/NR2B-ERK pathway in PC12 cells
Source: PLoS One. 2017 Sep 5;12(9):e0183702. doi: 10.1371/journal.pone.0183702 (PMC5584824; doi:10.1371/journal.pone.0183702)
Supplement: S9 File — (PDF) [file pone.0183702.s009.pdf]

SUPPORTING INFORMATION

fig.9a

NR2B			
**Control**	**GLU**	**GLU+LONC**	**LONC**
**0.40**	**1.10**	**0.53**	**0.08**
**0.56**	**1.06**	**0.52**	**0.16**
**0.55**	**1.16**	**0.56**	**0.15**
**0.49**	**1.08**	**0.60**	**0.10**

fig.9b

CaMKII			
**Control**	**GLU**	**GLU+LONC**	**LONC**
**0.96**	**1.14**	**0.73**	**0.16**
**0.86**	**1.20**	**0.62**	**0.12**
**0.95**	**1.26**	**0.66**	**0.10**
**1.05**	**1.28**	**0.54**	**0.08**

fig.9c

ERK1/2			
Control	GLU	GLU+LONC	LONC
0.50	0.50	0.53	0.58
0.56	0.56	0.54	0.56
0.52	0.56	0.56	0.55
0.50	0.58	0.49	0.50
P-ERK1/2			
Control	GLU	GLU+LONC	LONC
0.86	0.20	0.33	0.60
0.76	0.19	0.42	0.66
0.85	0.16	0.36	0.75
0.89	0.28	0.34	0.61
