# Supplementary material for: Longistyline C acts antidepressant in vivo and neuroprotection in vitro against glutamate-induced cytotoxicity by regulating NMDAR/NR2B-ERK pathway in PC12 cells
Source: PLoS One. 2017 Sep 5;12(9):e0183702. doi: 10.1371/journal.pone.0183702 (PMC5584824; doi:10.1371/journal.pone.0183702)
Supplement: S10 File — (PDF) [file pone.0183702.s010.pdf]

SUPPORTING INFORMATION

fig.10a

CREB			
Control	GLU	GLU+LONC	LONC
0.50	0.50	0.53	0.58
0.46	0.48	0.52	0.49
0.55	0.46	0.48	0.53
0.50	0.58	0.50	0.52

fig.10b

p-CREB			
Control	GLU	GLU+LONC	LONC
1.16	0.64	0.93	1.16
1.06	0.60	1.12	1.06
1.25	0.66	1.16	1.20
0.95	0.58	1.20	0.99
