# Supplementary material for: Longistyline C acts antidepressant in vivo and neuroprotection in vitro against glutamate-induced cytotoxicity by regulating NMDAR/NR2B-ERK pathway in PC12 cells
Source: PLoS One. 2017 Sep 5;12(9):e0183702. doi: 10.1371/journal.pone.0183702 (PMC5584824; doi:10.1371/journal.pone.0183702)
Supplement: S11 File — https://figshare.com/s/d99d1459759757174a66. DOI: 10.6084/m9.figshare.5331466. (PDF) [file pone.0183702.s011.pdf]

SUPPORTING INFORMATION

fig.11a

GADD153			
Control	GLU	GLU+LONC	LONC
0.30	1.80	0.63	0.47
0.26	1.78	0.72	0.44
0.25	1.66	0.56	0.30
0.20	1.58	0.64	0.56
0.89	1.28	0.71	0.70

fig.11b

GRP78			
Control	GLU	GLU+LONC	LONC
0.90	1.20	0.87	0.88
0.86	1.28	0.77	0.76
0.99	1.16	0.82	0.60

fig.11c

XBP-1			
Control	GLU	GLU+LONC	LONC
0.60	1.36	0.93	0.60
0.56	1.24	1.08	0.65
0.45	1.36	0.97	0.54

**0.50**	**1.38**	**0.93**	**0.62**

fig.11d

**caspase-12**			
**Control**	**GLU**	**GLU+LONC**	**LONC**
**0.30**	**0.84**	**0.35**	**0.18**
**0.22**	**0.83**	**0.27**	**0.23**
**0.26**	**0.74**	**0.37**	**0.24**
**0.26**	**0.84**	**0.25**	**0.17**

fig.11e

**caspase-9**			
**Control**	**GLU**	**GLU+LONC**	**LONC**
**0.20**	**1.20**	**0.33**	**0.27**
**0.26**	**1.19**	**0.32**	**0.22**
**0.25**	**0.96**	**0.46**	**0.30**
**0.20**	**1.28**	**0.44**	**0.31**
